# Role of Gut Microbiota on Cardio-Metabolic Parameters and Immunity in Coronary Artery Disease Patients with and without Type-2 Diabetes Mellitus

**DOI:** 10.3389/fmicb.2017.01936

**Published:** 2017-10-05

**Authors:** Lidia Sanchez-Alcoholado, Daniel Castellano-Castillo, Laura Jordán-Martínez, Isabel Moreno-Indias, Pilar Cardila-Cruz, Daniel Elena, Antonio J. Muñoz-Garcia, Maria I. Queipo-Ortuño, Manuel Jimenez-Navarro

**Affiliations:** ^1^Unidad de Gestión Clínica de Endocrinología y Nutrición, Instituto de Investigación Biomédica de Málaga, Hospital Universitario Virgen de la Victoria, Universidad de Málaga, Málaga, Spain; ^2^CIBER Fisiopatología de la Obesidad y Nutrición, Instituto de Salud Carlos III, Madrid, Spain; ^3^Unidad de Gestión Clínica Área del Corazón, Hospital Universitario Virgen de la Victoria, Instituto de Investigación Biomédica de Málaga, Universidad de Málaga, CIBERCV Enfermedades Cardiovasculares, Málaga, Spain

**Keywords:** gut microbiota, coronary artery disease, zonulin levels, gut permeability increase, TMAO production, factor Forkhead box P3, type-2 diabetes mellitus, anti-inflammatory IL-10

## Abstract

Gut microbiota composition has been reported as a factor linking host metabolism with the development of cardiovascular diseases (CVD) and intestinal immunity. Such gut microbiota has been shown to aggravate CVD by contributing to the production of trimethylamine *N*-oxide (TMAO), which is a pro-atherogenic compound. Treg cells expressing the transcription factor Forkhead box protein P3 (FoxP3) play an essential role in the regulation of immune responses to commensal microbiota and have an atheroprotective role. However, the aim of this study was to analyze the role of gut microbiota on cardio-metabolic parameters and immunity in coronary artery disease (CAD) patients with and without type-2 diabetes mellitus (DM2). The study included 16 coronary CAD-DM2 patients, and 16 age, sex, and BMI matched CAD patients without DM2 (CAD-NDM2). Fecal bacterial DNA was extracted and analyzed by sequencing in a GS Junior 454 platform followed by a bioinformatic analysis (QIIME and PICRUSt). The present study indicated that the diversity and composition of gut microbiota were different between the CAD-DM2 and CAD-NDM2 patients. The abundance of phylum *Bacteroidetes* was lower, whereas the phyla *Firmicutes* and *Proteobacteria* were higher in CAD-DM2 patients than those in the CAD-NDM2 group. CAD-DM2 patients had significantly less beneficial or commensal bacteria (such as *Faecalibacterium prausnitzii* and *Bacteroides fragilis*) and more opportunistic pathogens (such as *Enterobacteriaceae, Streptococcus*, and *Desulfovibrio).* Additionally, CAD-DM2 patients had significantly higher levels of plasma zonulin, TMAO, and IL-1B and significantly lower levels of IL-10 and FOXP3 mRNA expression than CAD-NDM2. Moreover, in the CAD-MD2 group, the increase in *Enterobacteriaceae* and the decrease in *Faecalibacterium prausnitzii* were significantly associated with the increase in serum TMAO levels, while the decrease in the abundance of *Bacteroides fragilis* was associated with the reduction in the FOXP3 mRNA expression, implicated in the development and function of Treg cells. These results suggest that the presence of DM2 is related to an impaired regulation of the immune system in CAD patients, mediated in part by the gut microbiota composition and functionality and the production and effects of their gut microbiota derived molecules.

## Introduction

Previous studies have shown an important connection between metabolism, intestinal microbial composition and the development of cardiovascular risk (Tremaroli and Backhed, 2012; Tang and Hazen, 2014; Org et al., 2015). Several authors have strongly supported the contribution of intestinal microbiota in the conversion of L-carnitine and dietary phosphatidylcholine in trimethylamine (TMA), which is absorbed into the bloodstream and then oxidized to trimethylamine *N*-oxide (TMAO) (a pro-atherogenic compound) by the hepatic enzyme flavin-containing monooxygenase-3 (Ufnal et al., 2015; Stremmel et al., 2017). Independent of traditional CVD risk factors, this microbiota-dependent metabolite TMAO has been related with the development of clinical CVD in the general population (Koeth et al., 2013; Tang et al., 2013, 2014, 2015; Wang et al., 2014; Troseid et al., 2015). Furthermore, elevated TMAO plasma levels are associated with increased CVD (Wang et al., 2011; Tang et al., 2013, 2014; Koeth et al., 2014).

Nevertheless, the intestinal bacterial species responsible for phosphatidylcholine breakdown have not yet been completely described, however, some species of *Bacteroidetes* (such as, *B*. *thetaiotaomicron* and *B*. *fragilis*) may be involved in the formation of TMA since they express phospholipases which hydrolyse dietary phosphatidylcholine to choline (Sitaraman, 2013). In addition, it has been reported that bacteria of the Erysipelotrichia class (*Firmicutes* phylum) can produce TMA from choline whereby it imitates a choline deficiency (Serino et al., 2014), while several families of bacteria such as *Deferribacteraceae, Anaeroplasmataceae, Prevotellaceae* (Koeth et al., 2013), and *Enterobacteriaceae* (Craciun and Balskus, 2012; Zhu et al., 2014) have been also associated with TMA/TMAO production.

On the other hand, TMAO has also been suggested to be a strong candidate molecule to mediate the development of type-2 diabetes mellitus (DM2) in animal and human studies (Kim et al., 2015). Moreover, coronary artery disease (CAD) is an important determining factor of the long term prognosis among patients with DM2 and the prevalence of diabetes in patients with CAD is up to 50% in many countries. Patients with diabetes have a 2- to 4-fold greater risk of developing atherosclerotic CAD than non-diabetic patients (Aronson and Edelman, 2016; Hölscher et al., 2016). Accumulating evidence suggests that DM2 and cardiovascular diseases such as hypertension, atherosclerosis, or heart failure are associated with alterations in the integrity of the gut barrier and augmented gut permeability (Ufnal and Pham, 2017).

Finally, gut microbiota has been reported to be a factor that is able to link intestinal immunity and host metabolism. It has been suggested that the increase or decrease of the abundant quantities of some specific bacteria may result in an increased generation of Tregs or in the reduced differentiation of pathogenic T cells, which may prevent inflammatory diseases (Smith et al., 2013). Treg cells expressing the transcription factor Forkhead box protein P3 (FoxP3) play an essential role in the regulation of immune responses to commensal microbiota. Furthermore, metabolites of commensal bacteria might regulate Treg development (Pereira et al., 2017). The administration of *Bifidobacterium infantis* 35624 has been found to induce Foxp3 T regulatory cells in human peripheral blood (Konieczna et al., 2012). It has also been described that FOXP3 positive regulatory T cells play a crucial role in maintaining the immune balance having an atheroprotective role (Hasib et al., 2016).

Therefore, the aim of this study was to analyze the role of gut microbiota on cardio-metabolic parameters and immunity in CAD patients with and without DM2. Furthermore, we have also analyzed the association of the gut microbiota found in these patients with the glucose metabolism, inflammatory status, intestinal permeability, and serum levels of TMAO and FOXP3 gene expression in peripheral blood.

## Materials and Methods

### Study Subjects

We included a total of 32 patients with CAD who were divided into two groups: 16 patients with DM2 (the CAD-DM2 group) and 16 patients without DM2 (the CAD-NDM2). The CAD group was defined by the presence of at least one coronary stenosis ≥50% of luminal diameter diagnosed by coronary angiogram. DM2 was defined by the history of diabetes diagnosed and/or the presence of treatment with medication (insulin or anti-diabetic drugs) and/or fasting blood glucose ≥126 mg/dl and/or glycated hemoglobin (HbA1c) ≥6.5%. Diabetic treatment of CAD patients with DM2 was the following: oral anti-diabetic (metformin *n* = 8 and glibenclamide *n* = 3) and insulin (*n* = 5).

All subjects underwent a complete physical examination, including measurements of height, weight, and blood pressure. The medical history of all patients was recorded, including the duration of diabetes, hypertension history, the history of taking anti-hypertensive and hypoglycemic drugs, and smoking history. The definition of systemic arterial hypertension was determined as a history of high blood pressure or treatment with anti-hypertensive medication. The definition of Dyslipidemia was determined as known but untreated dyslipidemia or currently undergoing treatment with lipid lowering medication. The family history of coronary heart disease was determined by interviewing the patients. The definition of a positive smoking history was determined as current smokers or cessation of smoking within the previous 3 months before the start of the study.

The following were considered as exclusion criteria: acute inflammatory disease, severe infective disease and/or cancer, chronic kidney disease (GFR < 45 mL/min/1.73 m^2^), hepatic impairment and autoimmune disease. This study was carried out in accordance with the recommendations of the Ethical Committee of the Virgen de la Victoria Hospital. Written informed consent was obtained in all cases in accordance with the Declaration of Helsinki. The protocol was approved by the Ethical Committee of the Virgen de la Victoria Hospital.

### Biochemical Variable Analysis

Serum was separated from blood samples which were collected after an overnight fast, aliquoted and immediately frozen at -80°C. Enzymatic methods (Randox Laboratories Ltd., United Kingdom) were employed to analyze the levels of serum cholesterol, triglycerides, HDL-cholesterol, glucose, and glycated hemoglobin (HbA1c) using a Dimension autoanalyzer (Dade Behring Inc., Deerfield, IL, United States). The Friedewald equation was used to calculate LDL-cholesterol. Standard enzymatic methods (Wako Bioproducts, Richmond, VA, United States) were used to measure gamma-glutamyl transpeptidase (GGT), glutamate-oxaloacetate transaminase (GOT), and glutamic pyruvic transaminase (GPT). RIA provided by BioSource S.A. (Nivelles, Belgium) was utilized for insulin quantification. The ELISA kit from BLK Diagnostics (Badalona, Spain) was employed to measure high sensitivity C-reactive protein (CRP) levels. To calculate the HOMA-IR we used the equation: HOMA-IR = fasting insulin (mIU/mL)/fasting glucose (mol/L)/22.5.

IL-10 and IL-1β cytokines were measured in serum samples using commercially available Novex^®^ ELISA Kits (Life Technology), performed according to the manufacturer’s instructions. The assay range was 7.8–500 pg/mL for IL-10 and 3.9–250 pg/mL for IL-1β.

### Anthropometric Measures

Standardized procedures were used to measure body weight, height, waist, and hip circumferences (Lohman et al., 1988). BMI was calculated as weight (kilograms) divided by height (in meters) squared.

### PCR Amplification and Analysis of 16S rDNA Sequences

DNA was extracted from fecal samples using the QIAamp DNA Stool Mini Kit (Qiagen, Hilden, Germany) following the manufacturer’s protocol. Amplification of genomic DNA was performed using barcoded primers that targeted the V2 to V3 regions of the bacterial 16S rRNA gene. Amplification, sequencing, and basic analysis were performed by using a GS Junior 454 platform according to the manufacturer’s protocols using a Titanium chemistry apparatus (Roche Applied Science, Indianapolis, IN, United States). For further details, see Moreno-Indias et al. (2015).

### Bioinformatic Analysis

Quantitative Insights into Microbial Ecology (QIIME) 1.8.0 software was used to analyze the 454-pyrosequencing data sets as previously described by our group (Moreno-Indias et al., 2015). Raw reads were filtered first following the 454 amplicon processing pipeline. The pyrosequencing reads were de-multiplexed and filtered further by means of the split_library.py script of QIIME. Reads with an average quality score lower than 25, ambiguous base calls, primer mismatches or shorter than 100 bp were excluded from the analysis in order to increase the level of accuracy. After the quality filter, the pipeline analysis used to analyze the 16S gene reads was the following: sequences were denoised and singletons excluded. The operational taxonomic units (OTUs) were selected by clustering sequences with a similarity of >97% and the representative sequences, selected as the most abundant in each cluster, were passed on to the UCLUST to obtain the taxonomy assignment and the relative abundance of each OTU by means of using the Greengenes 16S rRNA gene database. QIIME was used to evaluate alpha and beta diversity, as described (De Filippis et al., 2013).

### PICRUSt Analysis

The PICRUSt analysis was used to predict metagenome function by picking OTUs against the Greengenes database.^1^ As has been previously described by Bhute et al. (2016), “a closed reference based OTU picking approach was utilized to bin the amplicon sequences using latest Greengenes database 13.5 at 97% sequence similarity cut-off. The normalization for 16S rRNA gene copy number was carried out before prediction of the metagenome. This OTU table was used for predicting metagenome at different KEGG levels.” The R packages “pheatmap” were used for data analysis and plotting. Statistical analysis was done in R 3.3.3. *P*-values were corrected for multiple comparisons using the Benjamini–Hochberg method. A corrected *p* < 0.05 was considered as statistically significant.

### Intestinal Permeability

The plasma level of zonulin was determined by means of the enzyme linked immunosorbent assay (ELISA) using commercial kits (Immundiagnostik AG, Bensheim, Germany). Measurements were made in duplicate, and the mean values were used for the analysis. The detection limit for zonulin was established at 0.22 ng/mL. Intra- and inter-assay coefficients of variation were between 3 and 10%.

### Total RNA Extraction, cDNA Synthesis, and Real-Time Quantitative PCR (qRT-PCR)

The total RNA was extracted from peripheral blood mononuclear cells using TRIPURE Reagent (Sigma–Aldrich, United States) according to the manufacturer’s protocol. RNA was diluted in 20 μl RNase-free water and was subsequently quantified with a spectrophotometer (Nanodrop N-100, Thermo Scientific). Reverse transcriptions were performed using 1 μg of total RNA with the Transcriptor First Strand cDNA Synthesis Kit (Roche) and random hexamers in 20 μl reactions. The amplifications were performed by means of a MicroAmpH Optical 96-well reaction plate (Applied Biosystems, Foster City, CA, United States) on an ABI 7500 Fast Real-Time PCR System (Applied Biosystems, Foster City, CA, United States). Pre-validated and commercially available TaqMan^®^ primer/probe sets were used as follows: 18S rRNA (4319413E) used as the endogenous control for the target gene in each reaction and FOXP3 gene (RefSeq NM_001114377.1). A threshold cycle (*Ct* value) was obtained for each amplification curve and a *ΔCt* value was calculated first by subtracting the *Ct* value for human 18S rRNA cDNA from the *Ct* value for every sample and transcript. mRNA expression levels relative to 18S rRNA were calculated by means of the 2^-Δ^*^Ct^* method. All tests were performed in duplicate.

### Quantification TMAO in Serum Samples

Trimethylamine *N*-oxide was quantified in serum samples using Nuclear Magnetic Resonance (NMR) (Embade et al., 2016). For NMR analysis, serum samples were thawed for 30 min and aliquots of 300 μL were mixed with 300 μL of phosphate buffer (pH 7.0) containing 5 mmol/L TSP (Trimethylsilyl propionate) and 5% v/v D2O. The final mixtures were gently shaken and transferred to NMR 96 rack tubes. NMR spectra were measured at 300 K on a Bruker Avance IVDr 600 MHz spectrometer (Bruker Biospin, Germany) and a Sample Jet Robot (Bruker Biospin, Germany) was used for automatization of the measurements. For each sample, three complementary NMR spectra were recorded. A standard ^1^H spectrum with water suppression (NOESY) was assessed. We repeated the same experiment with an appended T2 relaxation filter implemented as a CPMG module to decrease broad signals from proteins and lipoproteins. Finally, a two dimensional ^1^H,^1^H JRES was measured to help with the identification of the metabolites. All spectra were acquired and processed within the TopSpin program (Bruker Biospin, Germany) applying an automatic phase correction and referenced against internal TSP (δ = 0.00 ppm). To identify and quantify the desired metabolites (TMAO) different amounts of these compounds were added to the serum samples, were measured by NMR and the values of the intensity peaks were represented against concentration. The pure metabolite molecules used for referencing were all obtained from Sigma–Aldrich (St. Louis, MO, United States). For all the spectra we measured the intensity of the peaks corresponding to TMAO (singlets) and the concentrations in the samples were calculated with the power fitted calibration curves.

### Statistical Analysis

Given that there are no previous studies assessing gut microbiota differences between patients with CAD with or without DM2, we have based our sample size estimation on a previous paper from Emoto et al. (2016). To calculate the sample size, we considered a difference in the mean relative abundance of *Bacteroidetes* between CAD patients and non-CAD controls with coronary risk factors of 0.084 and an estimated standard deviation between groups of 0.05, at least 9 patients in each study group were needed (90% power and a two-sided alpha level of 0.05). However, we increased the sample size to 16 samples per group.

The relative abundance of each OTU (taxa) were compared by a Wilcoxon test with a continuity correction using the Explicit software package specifically addressed to analyze microbiome data. α- and β-diversities were achieved by QIIME: α-diversity using a non-parametric *t*-test with a default number of Monte Carlo permutations of 999, and β-diversity using the ANOSIM statistical method with 99 permutations (Moreno-Indias et al., 2015). Continuous variables are summarized as mean ± SD. Discrete variables are presented as frequencies and percentages. Differences in clinical characteristics between two groups were analyzed using the Mann–Whitney *U* test. The Spearman correlation coefficients were calculated to estimate the correlations between variables. A lineal regression analysis was performed to identify individual bacteria as independent predictors for HOMA-IR, levels of inflammatory mediators, serum TMAO and plasma zonulin levels and relative expression of FOXP3 in the study groups. Statistical analyses were carried out with the statistical software package SPSS version 15.0 (SPSS Inc., Chicago, IL, United States). Values were considered to be statistically significant when the *p* < 0.05.

## Results

The general characteristics of the patients in both study groups are summarized in **Table 1**. There were no significant differences in age, sex, and body mass index (BMI) between the two study groups. The number of patients with dyslipidemia and hypertension was significantly higher in CAD-DM2 patients when compared with the CAD-NM2 patients. Triglyceride, glucose, and HbA1C were significantly higher and HDL-cholesterol was significantly lower in CAD-DM2 patients when compared with CAD-NM2 (*P* < 0.05). There was no significant difference in serum total cholesterol and LDL-cholesterol between the two study groups (*P* > 0.05). CAD-DM2 patients had significantly higher levels of inflammatory markers IL-1B and significantly lower levels of anti-inflammatory IL-10 when compared with non-diabetic patients. TMAO plasma levels were also significantly increased in CAD-DM2 patients when compared with CAD-NDM2 (*P* < 0.05). Finally, FOXP3 mRNA expression in PBMC was significantly lower (2.9-fold decrease) in CAD-DM2 patients when compared with CAD-NDM2 (*p* < 0.001).

**Table 1:** Biochemical, clinical characteristics, TMAO and inflammatory mediators serum levels and PBMC relative expression of FOXP3 in both study groups.

	CAD-DM2 *N* = 16	CAD-NDM2 *N* = 16	*P^∗^*
Age (years)	61.63 ± 9.15	58.75 ± 8.15	0.355
Gender, *n* (M/F)	13/3	13/3	0.652
BMI (kg/m^2^)	31.89 ± 6.04	29.23 ± 3.28	0.132
**Risk factors, *n* (%)**			
Hypertension	13 (81.25%)	7 (43.75%)	0.070
Dyslipidemia	10 (62.5%)	4 (25%)	0.075
Diabetes	16 (100%)	0 (0)	<0.001
Current smoking	13 (81.25%)	12 (75%)	0.999
CVA	1 (6.25%)	1 (6.25%)	0.715
**Medication, *n* (%)**			
Aspirin	10 (62.5%)	6 (37.5%)	0.289
Statin	7 (43.75%)	4 (25%)	0.457
ACEI/ARB	8 (50%)	6 (37.5%)	0.722
Beta-blocker	10 (62.5%)	8 (50%)	0.722
**Biochemical data**			
Total cholesterol (mg/dl)	179.0 ± 30.28	163.63 ± 29.05	0.153
HDL cholesterol (mg/dl)	28.68 ± 7.32	36.81 ± 8.68	0.008
LDL cholesterol (mg/dl)	104.7 ± 22.42	98.85 ± 23.51	0.192
Triglycerides (mg/dl)	200.37 ± 32.57	136.39 ± 35.9	0.001
HbA1c	7.70 ± 1.35	5.05 ± 0.56	0.001
Glucose (mg/dl)	153.40 ± 13.69	100.42 ± 16.02	0.001
HOMA-IR	9.91 ± 2.89	3.89 ± 0.98	0.001
GOT (U/l)	34.25 ± 6.70	30.21 ± 5.77	0.078
GPT (U/l)	45.0 ± 8.05	41.68 ± 6.60	0.135
GGT (U/l)	47.27 ± 16.00	44.20 ± 13.84	0.566
CRP (mg/L)	9.65 ± 1.59	9.50 ± 2.82	0.854
TMAO (ng/ml)	31.64 ± 8.5	16.26 ± 5.42	0.001
IL-1B (pg/ml)	120.41 ± 39.75	90.75 ± 37.23	0.037
IL-10 (pg/ml)	134.32 ± 36.24	162.31 ± 34.75	0.033
Foxp3 relative expression	0.27 ± 0.07	0.80 ± 0.15	<0.001

*BMI, body mass index; CVA, cerebrovascular accident; ACEI, angiotensin converting enzyme inhibitor; ARB, angiotensin II receptor blocker; LDL, low density lipoprotein; HDL, high density lipoprotein; HbA1c, glycated hemoglobin; GGT, gamma-glutamyl transferase; GOT, glutamic oxaloacetic transaminase; GPT, glutamic pyruvic transaminase, TMAO: trimethylamine *N*-oxide; L-1β, interleukin-1β; CRP, C-reactive protein; Foxp3, Forkhead box P3. Values are expressed as mean ± SD. ^∗^*P* < 0.05.*

### Analysis of the Diversity and Similarity of Microbial Communities in the Study Patients

A total of 249.815 good quality 16S rRNA gene sequences with an average of 11.895 ± 6527 sequences per sample passed the filters, which were applied by means of QIIME. In order to obtain a detailed structural overview of the microbiome of each subject who was enrolled in this study, an analysis of OTU was developed. The microbiota of all fecal samples after QIIME was composed by 2701 OTUS with a relative abundance higher than 1% in at least two samples (97% similarity cut-off).

Before assessing alpha and beta diversity measures, samples were rarefied to 3119 sequences, which represents the lowest number of quality reads which were obtained from any individual sample in the data set. The CAD-DM2 group had a lower number of OTUs than the CAD no-diabetic group (mean = 925 versus 987, *P* < 0.05, respectively).

The Chao index and Shannon index were calculated to estimate the alpha diversity. The average of community richness (Chao 1) and Shannon index of each group suggested a significant decrease in bacterial richness and diversity in fecal samples of CAD-DM2 with respect to CAD-NDM2 (*P* = 0.036 and *P* < 0.001, respectively) (**Table 2**). The rarefaction curve of the OTUs observed started to plateau at approximately 100 reads which suggests that a greater number of reads per sample would not have supplied a more extensive catalog of bacterial taxa.

**Table 2:** Estimate richness (Chao1) and diversity index (Shannon) indices among microbial communities obtained from fecal samples from CAD-DM2 and CAD-NDM2 patients.

	CAD-DM2 patients	CAD-NDM2 patients	*P^∗^*
	(*n* = 16)	(*n* = 16)	
Chao 1	374.01 ± 50.52	328.37 ± 66.20	0.036
Shannon	4.59 ± 0.68	5.90 ± 0.57	<0.001

*The richness estimator Chao and diversity estimator Shannon were calculated at 3% distance. ^∗^*P* < 0.05.*

**FIGURE 1:**
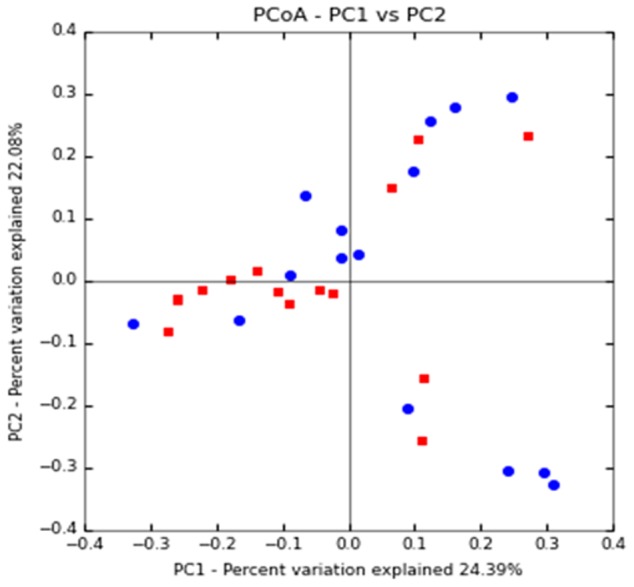
Clustering of fecal bacterial communities according to the different study groups by principal coordinate analysis (PCoA) using unweighted UniFrac distances. Each point corresponds to a community coded according to the patient group. The percentage of variation explained by the plotted principal coordinates is indicated on the axes. CAD-DM2 (red dots) and CAD-NDM2 (blue dots).

Principal coordinate analysis (PCoA) plots based on unweighted UniFrac metrics was used to evaluate the beta diversity. The present study showed that apart from a few samples from both study groups closer together in the ordination, a separation between the samples from the diabetic group and the non-diabetic group could be observed from PC1 and PC2 scores that accounted for 22.08 and 24.39% of total variations. ANOSIM with permutations suggested that the intestinal microflora composition of these two groups is significantly different (*P* = 0.039) (**Figure 1**).

### 16S rRNA Gene Pyrosequencing in Fecal Samples from CAD-DM2 and CAD-NDM2 Patients

**FIGURE 2:**
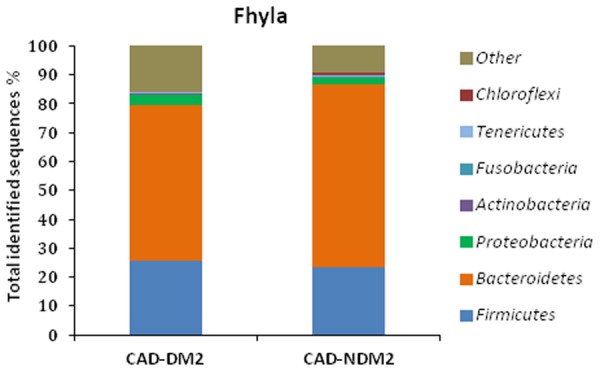
Pyrosequencing analysis of phyla in CAD-DM2 and CAD-NDM2 groups. Data are shown as a percentage of the total identified sequences per group.

In this study, variations in the composition of fecal microbiota of CAD-DM2 and CAD-NDM2 groups were observed at different bacterial levels. At the phyla level the majority of the OTUS were found to belong to *Bacteroidetes* (53.87% CAD-DM2 vs. 63.42% CAD-NDM2, *P* < 0.001), *Firmicutes* was the next most abundant (25.69% CAD-DM2 vs. 23.29% CAD-NDM2, *P* = 0.49) followed by *Proteobacterias* (3.67% CAD-DM2 vs. 2.05% CAD-NDM2, *P* = 0.04), but only *Bacteroidetes* and *Proteobacterias* exhibited significant differences between both, the CAD-DM2 and the CAD-NDM2 study groups. The remaining bacterial population belonged to the other four phyla (*Fusobacteria, Actinobacteria, Verrucomicrobia*, and *Tenericutes*) that had a relative abundance lower than 1% in at least two samples (**Figure 2**).

**FIGURE 3:**
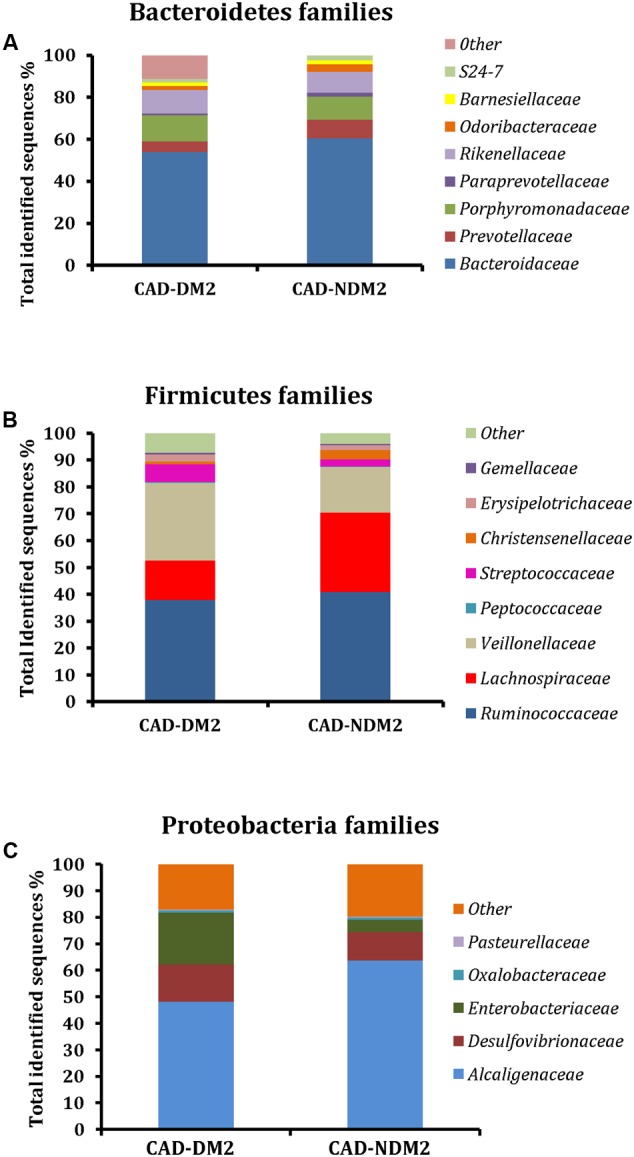
Microbial community structure at the family level using 454 sequence data in fecal samples of CAD-DM2 and CAD-NDM2 patients. Data are shown as a percentage of the total identified sequences per group. **(A)** Bacteroidetes families; **(B)** Firmicutes families and **(C)** Proteobacteria families.

Twenty-two families were detected among all groups. We found a significant increase in *Veillonellaceae* (28.93% CAD-DM2 vs. 16.97% CAD-NDM2, *P* < 0.001), *Enterobacteriaceae* (19.53% CAD-DM2 vs. 4.73% CAD-NDM2, *P* = 0.016), *Rikenellaceae* (11.13% CAD-DM2 vs. 9.92% CAD-NDM2, *P* = 0.023, *Streptococcaceae* (6.64% CAD-DM2 vs. 2.55% CAD-NDM2, *P* < 0.001), and *Desulfovibrionaceae* (13.95% CAD-DM2 vs. 10.69% CAD-NDM2, *P* < 0.001) in CAD-DM2 group. Furthermore, a significant decrease in the abundance was found in CAD-DM2 when compared with CAD-NDM2 for *Bacteroidaceae* (49.02% CAD-DM2 vs. 60.48% CAD-NDM2, *P* < 0.001), *Lachnospiraceae* (14.67% CAD-DM2 vs. 20.56% CAD-NDM2, *P* < 0.001), *Prevotellaceae* (5.06% CAD-DM2 vs. 8.85% CAD-NDM2, *P* = 0.05), *S24-7* (1.59% CAD-DM2 vs. 2.38% CAD-NDM2, *P* = 0.023, *P* < 0.001), *Odoribacteraceae* (1.90% CAD-DM2 vs. 3.57% CAD-NDM2, *P* = 0.02), and *Paraprevotellaceae* (0.79% CAD-DM2 vs. 1.81% CAD-NDM2, *P* < 0.05). In addition, no significant differences between the two study groups were found in other abundant families, such as *Ruminococcaceae* (37.87% CAD-DM2 vs. 40.91% CAD-NDM2, *P* > 0.05), *Alcaligenaceae* (48.10% CAD-DM2 vs. 63.63% CAD-NDM2, *P* > 0.05), *Porphyromonadaceae* (12.38% CAD-DM2 vs. 11.02% CAD-NDM2, *P* > 0.05), and *Erysipelotrichaceae* (2.63% CAD-DM2 vs. 1.75% CAD-NDM2, *P* > 0.05) (**Figure 3**).

**FIGURE 4:**
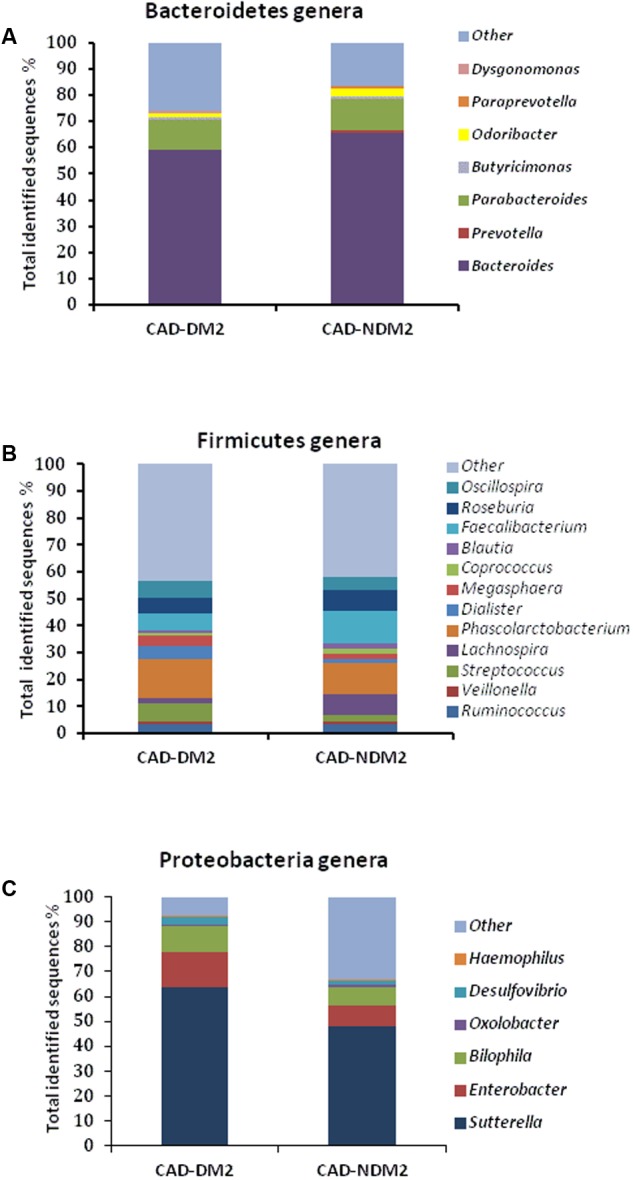
Relative abundance of predominant genera in the microbiota of CAD-DM2 and CAD-NDM2 patients. Data are shown as a percentage of the total identified sequences per group. **(A)** Bacteroidetes genera; **(B)** Firmicutes genera and **(C)** Proteobacteria genera.

Significant differences between the study groups were also found in the microbial composition at the genus level. A total of 40 genera were identified among the 32 fecal samples, with only significant differences in eight genera between the CAD-DM2 and the CAD-NDM2 groups. Thus, the genera *Enterobacter, Streptococcus, Dialister, Megasphaera*, and *Desulfovibrio* were significantly higher in the CAD-DM2 group when compared with the CAD-NDM2 group. On the other hand, we found in the CAD-NDM2 group a significant rise in the abundance of *Bacteroides, Faecalibacterium*, and *Prevotella* when compared with the CAD-DM2 patients. No significant differences in the abundance of *Sutterella, Phascolarctobacterium, Parabacteroides, Oscillospira, Roseburia, Ruminococcus, Odoribacter, Blautia*, and other minority genera were found between the two study groups (**Table 3** and **Figure 4**).

**Table 3:** Complete list of genus level microbial abundance (OTUs) in both study groups.

	Average bacteria abundance (OTUs) %		
Genus	CAD-DM2 *N* = 16	CAD-NDM2 *N* = 16	*P*^∗^
*Enterobacter*	14.25	8.29	0.03
*Streptococcus*	6.64	2.55	<0.001
*Dialister*	4.88	1.26	<0.001
*Megasphaera*	3.61	2.10	0.001
*Desulfovibrio*	3.23	1.85	0.001
*Bacteroides*	59.02	65.48	<0.001
*Faecalibacterium*	6.13	12.29	0.01
*Prevotella*	0.35	1.33	0.03
*Sutterella*	63.68	48.10	>0.05
*Phascolarctobacterium*	14.27	11.50	>0.05
*Parabacteroides*	12.20	11.55	>0.05
*Oscillospira*	6.39	5.10	>0.05
*Roseburia*	5.95	7.63	>0.05
*Ruminococcus*	3.28	3.51	>0.05
*Odoribacter*	1.43	2.72	>0.05
*Blautia*	1.05	2.12	>0.05

*Data are shown as a percentage of the total identified sequences per group. ^∗^*P* < 0.05.*

At a specie levels, we found a significant decrease in the abundance of *Bacteroides fragilis* (0.43% CAD-DM2 vs. 1.38% CAD-NDM2, *P* < 0.05), *Bacteroides ovatus* (0.99% CAD-DM2 vs. 1.86% CAD-NDM2, *P* < 0.05), and *Faecalibacterium prausnitzii* (2.03% CAD-DM2 vs. 3.72% CAD-NDM2, *P* < 0.05) in the CAD-DM2 group when compared with the CAD-NDM2 patients.

### Increased Circulating Zonulin Levels in CAD-DM2 Patients

The circulating zonulin levels were measured by means of the ELISA method. In the CAD-DM2 patients, circulating zonulin levels were significantly higher than in CAD-NDM2 patients (5.98 ± 1.4 vs. 3.34 ± 1.6 ng/ml, *P* < 0.001).

### Relationship between the Gut Microbiota Composition and Glucose Metabolism, Serum Levels of Inflammatory Mediators, Serum TMAO and Plasma Zonulin Levels and Relative Expression of FOXP3

In the CAD-DM2 patients, *Firmicutes* was positively correlated with HbA1c and fasting triglycerides (*r* = 0.849, *P* = 0.030, and *r* = 0.648, *P* = 0.043, respectively) and *Proteobacteria* was positively correlated with HOMA-IR (*r* = 0.754, *P* = 0.012), while *Bacteroidetes* was negatively associated with HOMA-IR (*r* = -0.849, *P* = 0.03). The abundance of *Streptococcus* was negatively associated with the serum HDL-cholesterol levels (*r* = -0.728, *P* = 0.017). We also confirmed a positive association between serum TMAO and the abundance of *Enterobacteriaceae* (*r* = 0.711, *P* = 0.021) and *Desulfovibrio* (*r* = 0.849, *P* = 0.03) and a negative association between TMAO and the abundance of the common gut commensals *Faecalibacterium prausnitzii* (*r* = -0.681, *P* = 0.030) and *Ruminococcaceae* (*r* = -0.699, *P* = 0.04). Likewise, the abundance of *Faecalibacterium prausnitzii* was negatively associated with plasma zonulin (*r* = -0.642, *P* = 0.045) and was positively associated with serum IL-10 levels (*r* = 0.663, *P* = 0.037). Finally, FOXP3 mRNA expression in PBMC was positively associated with the abundance of *Bacteroides fragilis* (*r* = 0.742, *P* = 0.037) and *Butyricimonas* (*r* = 0.717, *P* = 0.020).

In CAD-NDM2 patients, plasma zonulin levels were positively associated with the abundance of *Prevotella* (*r* = 0.681, *P* = 0.030) and *Rikenellaceae* (*r* = 0.669, *P* = 0.035), while serum TMAO was positively associated with *Bacteroides* (*r* = 0.742, *P* = 0.014) and negatively related to *Faecalibacterium prausnitzii* (*r* = -0.669, *P* = 0.035). Furthermore, there was a positive association between IL-10 and *Bifidobacterium* (*r* = 0.794, *P* = 0.006).

Subsequent lineal regression analysis including all the bacterial groups showed that the increase in *Proteobacterias* (*p* = 0.029, β = 0.548, *r*^2^= 0.83) was associated with the higher HOMA-IR found in CAD-DM2. On the other hand, in the CAD-DM2 group the increase in *Enterobacteriaceae* (*p* = 0.005, β = 0.945, *r*^2^= 0.96) and the decrease in *Faecalibacterium prausnitzii* (*p* = 0.041, β = -0.911, *r*^2^= 0.91) were associated with the increase in serum TMAO levels, while the decrease in the abundance of *Bacteroides fragilis* (*p* = 0.039, β = 0.911, *r*^2^= 0.91) was associated with the reduction in the FOXP3 mRNA expression.

### Functional Differences in Gut Microbiota between Both Study Groups

The PICRUSt analysis indicated that the genes for the metabolism of carbohydrates, lipids, amino acids, and energy were significantly over represented in CAD-NDM2 in comparision with CAD-DM2 (*P* < 0.05). Moreover, in the microbiota of CAD-NDM2 patients, we found inside the carbohydrate metabolism, significantly enriched for the proportion of genes related to glycolysis/gluconeogénesis (*P* = 0.036), pyruvate metabolism (*P* = 0.029), inositol phosphate metabolism (*P* = 0.027), pentose phosphate pathway (*P* = 0.016), propanoate metabolism (*P* = 0.020), butanoate metabolism (*P* = 0.023), and starch and sucrose metabolism (*P* = 0.016), additionally, in the lipid metabolism pathways we observed an increase in the proportions of genes for fatty acid biosynthesis (*P* = 0.013), fatty acid metabolism (*P* = 0.021), glycerophospholipid metabolism (*P* = 0.036), sphingolipid metabolism (*P* = 0.008), biosynthesis of unsaturated fatty acids (*P* = 0.020), and secondary bile acid metabolism (*P* = 0.005).

Furthermore, the metagenomic comparison between CAD-DM2 and CAD-NDM2 groups showed that gene families linked to amino acid metabolism [including the metabolism of glycine (*P* = 0.022), alanine (*P* = 0.043), and glutamine (*P* = 0.029)] and energy metabolism such as sulfur (*P* = 0.036) and nitrogen metabolism (*P* = 0.016) were significantly depleted in the CAD-DM2 group.

**FIGURE 5:**
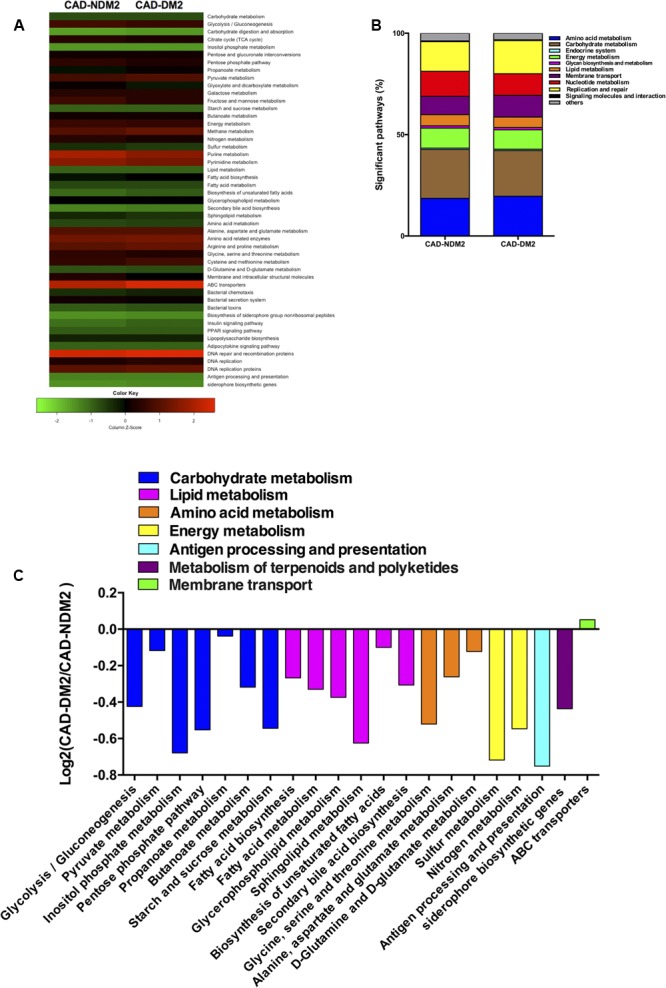
Predicted functional composition of metagenomes based on 16S rRNA gene sequencing data of CAD-DM2 and CAD-NDM2 groups. **(A)** Heatmap of differentially abundant KEGG pathways identified in both study groups. The values of color in the heatmap represent the normalized relative abundance of KEGG pathways. **(B)** Relative abundances of significant predicted functions of each study group. **(C)** Log2-fold change of the abundances of KEGG categories showing significant difference between CAD-DM2 and CAD-NDM2 groups.

Finally, the microbiota from CAD-NDM2 patients were also significantly more enriched with genes for antigen processing and presentation (*P* = 0.006) and siderophore biosynthesis (*P* = 0.036) when compared with CAD-DM2 patients (**Figure 5**).

## Discussion

The present study in which we used 16S ribosomal RNA of gut microbiota for high throughput sequencing has demonstrated that the diversity and gut microbial composition were different between CAD-DM2 and CAD-NDM2. We also observed that the CAD-DM2 patients had significantly higher levels of zonulin, TMAO, and IL-1B and significantly lower levels of IL-10 and FOXP3 mRNA expression than CAD-NDM2.

Microbiota diversity is essential to maintain ecosystem stability and efficiency. The α-diversity analysis in this study showed that the Shannon and Chao 1 indices were significantly lower in the CAD-DM2 patient group when compared with the CAD-NDM2 group. These data revealed that bacterial communities in diabetic patients with CAD had lower taxa richness than those in the non-diabetic group. These results may suggest that the decrease in gut microbiota diversity in CAD-DM2 patients might be related to the presence of DM2. In addition, the unweighted UniFrac PCoA plot analysis indicated a clustering of the microbial populations of the CAD-DM2 patients away from that of the CAD-NDM2, confirming that the presence of DM2 in patients with CAD can significantly alter intestinal microbial populations.

On the other hand, the taxonomy based analysis of the assigned sequences showed that the presence of DM2 in patients from CAD is related to a decrease in the phylum *Bacteroidetes* and an increase in the phyla *Firmicutes* and *Proteobacteria*. Moreover, at genera level the CAD-DM2 patients had more opportunistic pathogens such as *Enterobacteriaceae, Megasphaera, Streptococcus, Dialister*, and *Desulfovibrio*, and lower beneficial or commensal bacteria including *Bacteroides* (*Bacteroides fragilis*), *Faecalibacterium (Faecalibacterium prausnitzii*), and *Prevotella* when compared with CAD-NDM2. Previous studies using terminal restriction fragment length polymorphism (T-RFLP) analysis demonstrated a decrease in the phylum *Bacteroidetes* and an increase in the phylum *Firmicutes* in CAD patients when compared with healthy subjects. Moreover, they observed that the order Lactobacillales, in which *Streptococcus* genus is included, was significantly increased in the CAD group when compared with healthy subjects (Emoto et al., 2016, 2017). Cui et al. (2017) revealed that the phyla *Bacteroidetes* and *Proteobacteria* were decreased, while the phyla *Firmicutes* and *Fusobacteria* were increased in coronary heart disease patients when compared with healthy controls by high throughput sequencing. In our study, the significant decrease in the phylum *Bacteroidetes* and the increase in the abundance of the phylum *Proteobacteria* in the CAD-DM2 group possibly reflected the fact of suffering from DM2, one of the major risk factors for CAD.

After using PICRUSt, we were able to predict that the gut microbiome of CAD-DM2 patients showed a depletion in genes involved in metabolic functions such as the metabolism of carbohydrates, lipids, amino acids, and energy as well as in genes of antigen processing and presentation related to inflammation and immune response and siderophore biosynthesis. The enrichment in this siderophore pathway could suggest a higher intra- or inter-species communication in CAD-NDM2 patients, due to the fact that siderophore act as quorum sensing molecules for gram-negative organisms (McHardy et al., 2013). These results indicate that the presence of DM2 in CAD patients may also influence the functional diversity.

Koren et al. (2011) compared the microbiota among the gut, oral cavities, and the atherosclerotic plaque in patients with aterosclerosis. This study suggested that the bacteria which is present in the atherosclerotic plaque may be derived from the gut or the oral cavities (Koren et al., 2011). Furthermore, the gut microbiota is essential to bioconvert cholesterol into bile acids, which are necessary for the excretion and the absorption of cholesterol (Fagerberg et al., 2010). Thus, the significantly higher levels of *Streptococcus* (a common oral and gut taxón) found in CAD-DM2 patients when compared with CAD-NDM2 patients, which were negatively correlated with HDL cholesterol levels in these diabetic patients, could be influencing CAD evolution through the regulation of the lipid metabolism in the host.

The present study has demonstrated that CAD-DM2 patients had significantly higher plasma levels of TMAO when compared with CAD-NDM2. Tang et al. (2015) also found that the presence of diabetes in those patients belonging to a heart failure cohort was associated with elevations in plasma TMAO which were statistically relevant. Moreover, Gao et al. (2014) described that dietary TMAO supplementation in mice increases impaired glucose tolerance, insulin signaling, and promotes the inflammation of adipose tissue. Additionally, we found that the presence of certain specific bacterial taxa in human feces was associated with the concentration of plasma TMAO in both study groups. We observed that the plasma TMAO concentrations were significantly and negatively associated with the abundance of *Faecalibacterium prausnitzii* in CAD-DM2 patients. Moreover, we found a significant positive correlation of plasma TMAO concentrations with *Enterobacteriaceae* and *Desulfovibrio.* According to our results, other human and animal studies have suggested that several families of bacteria are involved in the production of TMA/TMAO such as *Prevotellaceae* (Koeth et al., 2013) and *Enterobacteriaceae* (Craciun and Balskus, 2012; Zhu et al., 2014). Additionally, it has been previously reported that the increase in the conversion of choline to TMA can be caused by the expression of the *cutC* gene by bacteria such as *Desulfovibrio* (Craciun and Balskus, 2012).

On the other hand, in order to enter the bloodstream, the microbiota derived molecules TMAO need to pass the gut-blood barrier. Accordingly, we observed that plasma zonulin levels were significantly higher in CAD-DM2 patients. Recent research has revealed that circulating zonulin levels are significantly higher in patients with diabetes, polycystic ovary syndrome, obesity, non-alcoholic fatty liver disease, all of which are regarded as traditional risk factors of atherosclerosis (Li et al., 2016).

Therefore, in our study, CAD-DM2 patients presented an alteration in gut microbiota equilibrium (dysbiosis), a disruption of gut barrier function and an increase in gut permeability which altogether may result in aberrant production and absorption of microbe derived metabolites such as TMAO, which can exert their atherogenic effect through alterations in cholesterol and bile acid metabolism, activation of inflammatory pathways, and promotion of foam cells formation. This situation could contribute to an increased risk of major adverse cardiovascular events and death in the CAD-DM2 group.

The pathological basis of CAD is aterosclerosis. Furthermore, inflammation plays a decisive role in atherosclerotic plaque progression, plaque rupture, and thrombosis, which are the initial factors in acute coronary syndrome (Min et al., 2017). In addition, intestinal permeability may contribute to CAD through the production of inflammatory cytokines and the weakening of the stability of plaque. Thus, in this study the significantly higher serum IL-1B levels generated in the gut epithelium during the systematic or localized inflammation in CAD-DM2 patients could also raise intestinal permeability and facilitate intestinal translocation of microbial components and metabolites into the circulation. Moreover, IL-1β enhance cholesterol uptake from human macrophages by upregulation of their oxidized LDL receptors, resulting in foam cell formation and plaque growth (Ikonomidis et al., 1999). In a previous study, IL-1β levels were associated with a less favorable prognosis after acute coronary syndrome (Van Tassell et al., 2013).

Additionally, the significant decrease in the peripheral FOXP3 mRNA expression and serum IL-10 in the CAD-DM2 when compared with the CAD-NDM2 group was significatively associated with the decrease in the abundance of *Bacteroides fragilis* and *Faecalibacterium prausnitzii*, respectively. *Bacteroides fragilis* plays an important role in mucosal T cell homeostasis by means of regulating the function of T cells (Mazmanian et al., 2008). The immunomodulatory molecule polysaccharide A derived from the human commensal *Bacteroides fragilis* mediates the conversion of CD4+ T cells into Foxp3+ Treg cells that produce IL-10 during commensal colonization, stimulating immunological development within mammalian hosts (Round and Mazmanian, 2010). At the same time, IL-10 is a key cytokine in Treg-mediated suppression (Wang et al., 2001). It has been described as a decreased percentage of peripheral CD4 + CD25 + Foxp3 + Treg and serum IL-10 level in patients with DM2. Furthermore, the percentages of peripheral CD4 + CD25 + Foxp3 + Treg and serum IL-10 level were influenced by diabetic complications such as cardiovascular diseases (Qiao et al., 2016).

On the other hand, the commensal bacteria *Faecalibacterium prausnitzii* is an important supplier of butyrate to the colonic epithelium. Butyrate is a short chain fatty acid (SCFA) that promotes the integrity of gut epithelial tight junctions as well as increases the release of the anti-inflammatory and anti-atherogenic cytokine IL-10 (Caligiuri et al., 2003) and promotes Foxp3 + Treg induction (Furusawa et al., 2013). So, the significant reduction in the abundance of *Faecalibacterium prausnitzii* and in the predicted proportion of genes related to the butanoate metabolism that we found in the CAD-DM2 patients when compared with CAD-NDM2 may partially explain the significantly lower levels of zonulin and serum IL-10 and the significantly higher levels of serum IL-1β and plasma TMAO in the CAD-DM2 patients.

Our study has certain limitations but also some important strengths. The limitations include the number of patients which was small and the possible influence of conflicting factors such as diabetes medication over gut microbiota. The strengths of our study lie in the well matched cohorts and the next generation sequencing of the microbiome.

## Conclusion

We have demonstrated that the presence of DM2 in CAD patients is associated with a gut microbiota change and reduced diversity together with functional metagenomic differences. CAD-DM2 patients had less beneficial or commensal bacteria (such as *Faecalibacterium prausnitzii*, and *Bacteroides fragilis*) and more opportunistic pathogens (such as *Enterobacteriaceae, Streptococcus*, and *Desulfovibrio)* that may impair intestinal barrier function (increasing zonulin levels), enhance the serum levels of TMAO, and may therefore contribute to inflammatory processes related to CAD by means of increasing the production of inflammatory cytokines (such as IL-1B). Moreover, the significant reduction in the abundance of *Faecalibacterium prausnitzii* and *Bacteroides fragilis* found in CAD-DM2 patients was associated with the decrease in the release of the anti-inflammatory and anti-atherogenic cytokine IL-10 and the peripheral FOXP3 mRNA expression, respectively, both implicated in the development and function of Treg cells. These results suggest that the presence of DM2 is related to an impaired regulation of the immune system in CAD patients, mediated in part by the gut microbiota composition and functionality and the production and effects of their gut microbiota derived molecules. Thus, gut microbiota may represent a new target for therapeutic manipulation, treatment, and prevention of complex cardiometabolic diseases through regulation of the immune system.

## Author Contributions

MQ-O, AM-G, and MJ-N: Conceived the study and developed the experimental design. LJ-M, PC-C, DE, AM-G, MQ-O, and MJ-N: Were responsible for the acquisition and selection of all samples utilized in this study. LS-A, DC-C, IM-I, and MQ-O: Performed all laboratory assays. LS-A, IM-I, DC-C, MJ-N, and MQ-O: Compiled the database and performed the statistical analysis and data interpretation. LS-A, DC-C, IM-I, LJ-M, PC-C, DE, MJ-N, and MQ-O: Wrote the paper. LJ-M, PC-C, AM-G, MJ-N, and MQ-O: Provided critical revision. All authors read and approved the final manuscript.

## Conflict of Interest Statement

The authors declare that the research was conducted in the absence of any commercial or financial relationships that could be construed as a potential conflict of interest.

## Footnotes

1 http://huttenhower.sph.harvard.edu/galaxy/
